# Critical Evaluation of Global Infection Prevention and Control Guidelines for Dentistry Published during the First 2 Years of the COVID-19 Pandemic

**DOI:** 10.1155/2024/6611105

**Published:** 2024-06-06

**Authors:** Khlood Alkurdi, Rowaina Mansouri, Aseel Ismail, Noha Seoudi

**Affiliations:** ^1^ Institute of Dentistry Queen Mary University of London, London E1 2AD, UK; ^2^ Ministry of Education, Riyadh, Saudi Arabia; ^3^ Faculty of Dentistry King Abdulaziz University, Jeddah, Saudi Arabia; ^4^ College of Medicine and Dentistry Ulster University, London, UK; ^5^ Cairo University, Cairo, Egypt

## Abstract

**Objectives:**

The outbreak of the coronavirus disease (COVID-19) encouraged immediate actions by governments and healthcare associations across the world to flatten the curve and prevent health systems from being overburdened. As dentistry comprises aerosol-generating procedures (AGPs), which could increase the risk of infection, various guidelines were issued for dental services which focused on infection prevention and control (IPC) measures for COVID-19. This systematic review focuses on dental IPC guidelines, with the aim of comparing these guidelines against a gold standard.

**Method:**

The Preferred Reporting Items for Systematic Reviews and Meta-Analyses (PRISMA) 2020 checklist was employed. Predefined inclusion and exclusion criteria were constructed. Information sources comprised Google Scholar, PubMed, and a manual search from December 2019 to December 2021. The Appraisal of Guidelines for Research and Evaluation (AGREE II) instrument was used. Consensus scoring was applied for all guidelines.

**Results:**

A total 61 guidelines were included in the review. The UK national guideline was used as a gold standard as it ranked the highest AGREE score (75 out of 84) and thus was established for comparison with each of the included guidelines. Overall, 40% of the included guidelines had a high consensus score in relation to the UK national guideline.

**Conclusion:**

This systematic review highlighted the variability in content and quality of advice given by different organizations in response to the COVID-19 pandemic in their efforts to reduce SARS-CoV-2 transmission in dentistry. Establishing a single worldwide fast-acting dental organization would ensure that high-quality standardized guidance is available, to enhance health equality and worldwide dental clinical standards.

## 1. Introduction

Since the outbreak of the coronavirus disease (COVID-19) in December 2019, an estimation of 6,792,156 mortalities has been recorded worldwide, as of 22 February 2023 [1]. COVID-19 immediately became a public health concern on a worldwide scale and was declared a pandemic by the World Health Organization (WHO) in March 2020 and the Public Health Emergency of International Concern (PHEIC) [2].

This zoonotic infectious virus is caused by severe acute respiratory syndrome coronavirus 2 (SARS-CoV-2), and its mode of transmission is through direct interaction, droplet, and potential aerosol transmissions, where it can be carried for 3 hr and can attach to hard surfaces for a few days. This makes it extremely infectious [3]. Incubating asymptomatically in the host for a maximum of 2 weeks, COVID-19 may cause respiratory failure, and patients often present with fever, fatigue, and severe dry cough accompanied by dyspnea [3]. Additionally, it has proven aggressively fatal to medically compromised patients and the elderly and has presented with vertical transition from mother to newborn [4].

Given the COVID-19's mode of transmission, dentistry was considered one of the professions that carried a high risk of it spreading among both dental healthcare workers (DHCW) and patients. Research has proven that saliva is a potential harbor of this virus, and it can carry it for 29 days [5]. Furthermore, most routine dental treatments are aerosol-generating procedures (AGPs), meaning that devices used in these procedures, such as high-speed motor and ultrasonic scalers, are able to generate aerosols of very small particles measuring less than 5 *μ*m [6]. This conveys the necessity for dentistry-specific transmission-based infection prevention and control (IPC) precautions in the different dental settings (dental hospitals, outpatient clinics, and community dental clinics).

While treatments and vaccines were still in the process of development, most countries restricted the provision of dental care to urgent and emergency treatments only. In the USA, 198,000 dental clinics were closed [7]. Routine dental services were curtailed, either because of the lack of personal protective equipment (PPE) or to assist in flattening the curve, to safeguard patients as well as DHCWs. In the UK, only urgent dental care was provided in the period between March and June 2020 in special centers. Additional safety measures were implemented, such as telephone risk assessment and triaging to decide if a face-to-face appointment was required. When it was deemed necessary to provide face-to-face urgent/emergency dental care, patients were booked to attend one of the urgent dental care centers where strict IPC measures were implemented, including recording body temperatures, using transmission-based IPC precautions with the right level of PPE, and reducing aerosol generation by using a rubber dam, four-handed dentistry, and high-volume suction. Furthermore, a meticulous cleaning and disinfecting regimen was implemented [8].

As international authorities approved the reopening of community dental care services after lockdown, various guidelines were published on IPC recommendations for dental healthcare services. This is because, according to the WHO, IPC is a scientific method and a practical option designed to protect patients and healthcare workers from infection-related harm. It plays an important role in infectious illness prevention [9]. These IPC guidelines were published by different organizations in different countries throughout the whole world. Additionally, a few of them had published multiple versions throughout the pandemic, implementing best evidence as it emerged. Consequently, DHCWs in different parts of the world followed diverse protocols, which led to variability in practice in different countries, compromising health equality and standards.

Assessing the quality of all dental IPC guidelines published during the first two waves of the COVID-19 pandemic is a very important reflective process to help guideline developers plan a universal strategy for future similar emergency situations. The implementation of standardized and evidence-based IPC protocols in dental healthcare services is crucial to improving patient and healthcare worker safety and advancing global health equity. This systematic review aims to answer the following question: “With regard to the IPC guidelines in dentistry published during the first 2 years of the COVID-19 pandemic, what is the level of consensus of advice given by different organizations worldwide compared to the highest standard guideline according to the AGREE II tool?”.

## 2. Methods

### 2.1. Eligibility Criteria

Guidelines were retrieved based on a previously established eligibility criterion. The inclusion criteria were as follows: the latest version of guidelines/recommendations related to IPC during COVID-19 in dental settings. The search framework included publications from December 2019 to December 2021 and in the English language. The exclusion criteria were as follows: any other COVID-19 guideline not related to IPC or dentistry, duplicates, systematic reviews, dissertations, letters, personal opinions, book chapters, conference abstracts, and pilot studies. Additionally, if updates of a guideline were present, older versions were excluded to ensure accurate comparison with guidelines published late. This approach helps maintain focus on the most recent information in COVID-19 and avoids confusion with earlier versions.

### 2.2. Information Source and Search Strategy

(1) Literature search: An electronic search was done systematically using online scientific servers (Google Scholar and PubMed) to retrieve relevant guidelines and recommendations on COVID-19 IPC in dentistry. The search was conducted from December 2019 to December 2021. An additional manual search was performed for the following websites: the National Health Service (NHS), Public Health England, Scottish Dental Clinical Effectiveness Programme, the Faculty of General Dental Practice, the College of General Dentistry, the Royal College of Surgeons of Edinburgh, the WHO, the US dental guidelines, Centers for Disease Control and Prevention, the Chinese Stomatological Association, and the Canadian Dental Association.

(2) Keywords and search terms: (guidelines OR SOP OR (standard operating procedures) OR recommendations OR consensus) AND (COVID-19 OR coronavirus OR pandemic OR SARS-CoV-2) AND (IPC OR infection prevention control) OR (cross-infection) OR (infection prevention) AND dentistry OR (dental hospital) OR (general dental practice) OR (dental surgery) OR (dental clinic).

### 2.3. Selection Process

Titles and abstracts of all retrieved papers were assessed independently by two researchers (AI and KA). When there was a dispute, discussion was used to reach a decision. A third author (RM) was involved to assist in reaching consensus when required. Subsequently, full-text papers were independently evaluated for inclusion by the same two researchers (AI and KA). Consensus on eligibility criteria was obtained through discussion, and, if necessary, a third author (RM) was engaged.

### 2.4. Data Collection Process and Data Items

After the selection process, the guidelines went through an evaluation and data extraction process. Three authors (AI, KA, and RM) used a data extraction sheet created in Microsoft® Excel (version 16. 69.1) to gather information from eligible guidelines. This step was independently done in three pairs (KA–RM, RM–AI, and KA–AI). Extracted data were then compared, and any differences were discussed, and consensus was reached. All retrieved guidelines were unambiguous; therefore, it was unnecessary to contact the issuing authorities of the guidelines to get more information.

(1) Outcome measures: For each guideline, qualitative data were extracted—ame of publishing organization, date of publication, country, definition of AGP and non-AGP, fallow time, protocol for PPE in regard to AGP and non-AGP, fallow time after AGP, ventilation requirement, triaging recommendation, telephone clinic, protocol for risk assessment for both staff and patients, measures for vulnerable patients, hard surface disinfection regimen, level of hand hygiene, type of respirator recommended, the protocol for positive COVID-19 patients, and test requirements for staff and patients as well as the vaccination recommendation for staff. Data extraction was performed by the same three pairs of reviewers. Any discrepancies in decisions or justifications for judgements were solved by consensus.

(2) Guideline identification: Each included guideline was assigned a numbered code to facilitate convenient identification and recognition across all 61 guidelines. Coding numbers are presented in Table 1.

### 2.5. Study Risk of Bias and Quality Assessment

Risk of bias was assessed in the included studies using the Appraisal of Guidelines for Research and Evaluation (AGREE II) instrument. AGREE II addresses six domains: (1) scope and purpose; (2) stakeholder involvement; (3) rigor of development; (4) clarity of presentation; (5) applicability; and (6) editorial independence. The same pairs of researches who performed the data extraction applied the tool to each included guideline and documented the justifications for judgements of scores that ranged from 1 to 4: 1, strongly disagree; 2, disagree; 3, agree; and 4, strongly agree.

### 2.6. Synthesis Methods

The interrater reliability kappa value was used at this stage to calculate the percentage of agreement between authors in the Cohen's kappa guideline. Furthermore, the Saphiro–Wilk normality test was used to detect if data were normally distributed or not.

### 2.7. Consensus Score and Identification of the Gold Standard

After critically appraising each recommendation, the guideline with the highest AGREE II score was represented as the gold standard and acted as the benchmark for consensus guideline comparisons. The consensus scoring was constructed to yield information on guideline grading in relation to the presence of extracted data, in comparison to the gold standard (guidelines consensus). The maximum scoring was 19 points, as each of the data extraction domains was given one point. Furthermore, consensus between guidelines in each domain was calculated (recommendation consensus).

## 3. Results

### 3.1. Selected Guidelines

**Figure 1:**
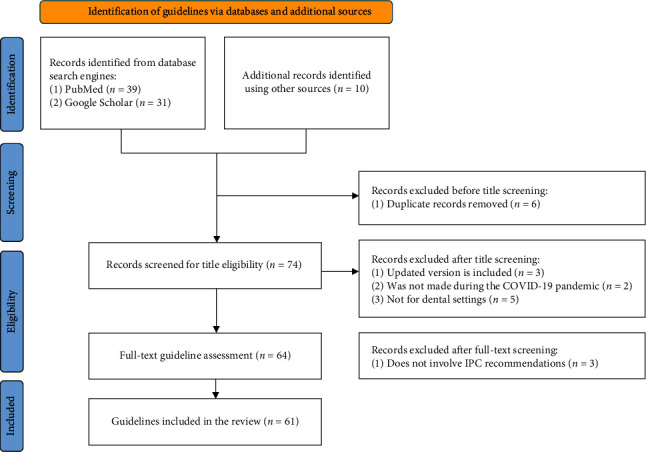
PRISMA 2020 flowchart illustrating the stages of literature search and screening.

A total of 39 guidelines were retrieved from PubMed and 31 from Google Scholar. An additional 10 guidelines were identified using a manual search. Of these, six guidelines were excluded because they were duplicated, to yield 74 guidelines. Two screen stages (title and text) were made to evaluate consistency with the eligibility criteria and to retrieve eligible data. No abstract screen was conducted as not all guidelines included an abstract or summary. When titles were screened, 13 guidelines did not meet the inclusion criteria and were excluded. The final number of included guidelines was 61 (for guidelines references, see Table 1). The identification process is demonstrated in the PRISMA 2020 flowchart in Figure 1.

### 3.2. Risk of Bias and Quality Assessment

The AGREE II critical appraisal tool was used for risk of bias and quality assessment of each guideline. It consists of 23 questions, with answers rated from 1 to 4, thus yielding a maximum score of 92 for each guideline. A summary table of these assessments is provided in Table 1. The author interrater reliability kappa value was 0.82, and the percentage of agreement was 94.04%, which is regarded as “almost perfect or perfect agreement” in the Cohen's kappa guideline [10].

**Figure 2:**
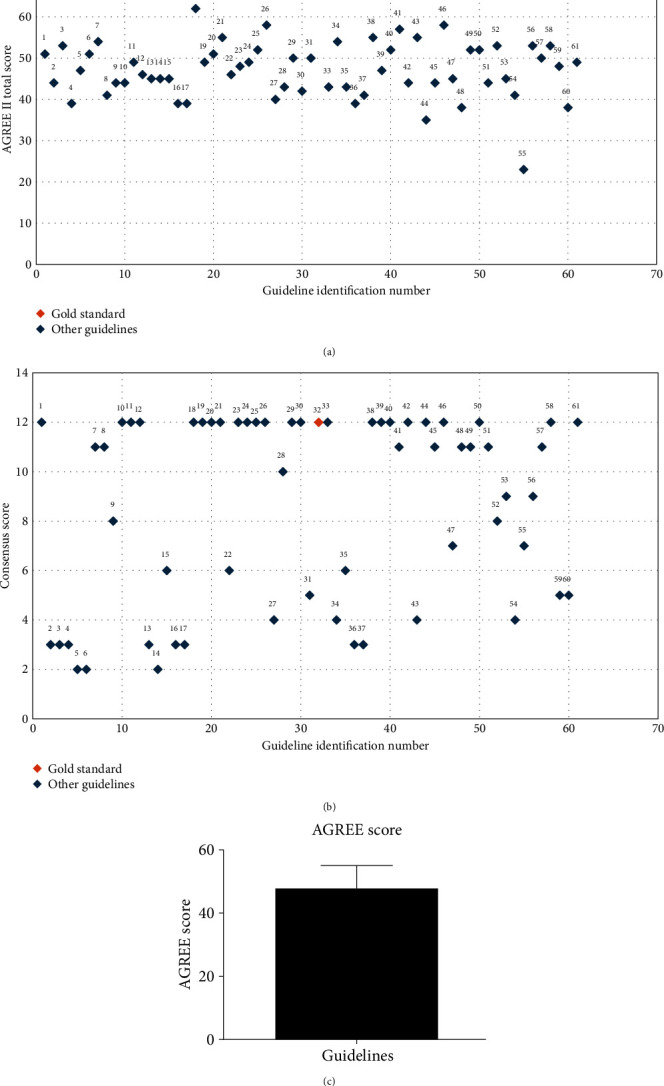
(a) AGREE II scoring for all guidelines included in this study. Guidelines are identified according to the number assigned in Table 1. (b) Consensus scoring for all guidelines included in this study.(c) Mean and SD scores for all included guidelines.

The UK-wide guidelines were published unitedly, but England, Scotland, Wales, and Northern Ireland (2020) scored the highest AGREE score (75 out of 92). For that reason, it was used as the gold standard to be compared with each of the included guidelines in this systematic review. A scatter plot in Figure 2(a) demonstrates the AGREE II score of each guideline. The total AGREE score of each guideline was analyzed using the PRISM Statistics software (GraphPad, USA), which can be seen in Figure 2(c). Additionally, the AGREE II score for all guidelines passed the KS normality test and was shown to be normally distributed. The mean was shown to be 47.41 ± 7.546 standard deviation (SD).

### 3.3. Key Characteristics of the Gold Standard

To facilitate accurate comparison within guidelines and against the gold standard, data were first extracted for the gold standard on all 19 domains (Table 2). Interestingly, even the gold standard did not show 100% completeness of all domains, and it lacked some information, such as the definition of non-AGP and regulations on telephone clinics.

### 3.4. Total Consensus with the Gold Standard

Records revealed 40% of recommendations being in line with the identified benchmark with regard to consensus scoring. All other recommendations fell below that level. This is illustrated in Table 1, which comprises the total consensus score for each guideline, and was used to generate a consensus scatter plot, presented in Figure 2(b).

### 3.5. Domain Consensus with the Gold Standard

Only 24 out of the 61 guidelines were in line with the benchmark as the other guidelines showed variability in recommendations. Table 2 presents the detailed domains' consensus scores along with the main reasons for variation. In the domain focused on the protocol of PPE, 13 guidelines did not mention information on donning and doffing, while 48 contained this information. Additionally, 27 guidelines emphasized the importance of staff risk assessment, while 34 did not mention its protocols. Furthermore, hand hygiene was referred to in 37 guidelines out of the 61, and the recommended type of respirator was discussed in only 44 guidelines.

Additionally, divergence within the guidelines was noted. There was variability in the size of aerosols defined in AGP procedures, as some guidelines, including the gold standard, approved particle size of 5 *μ*m, while others accepted 10 *μ*m or even 50 *μ*m. Additionally, only a third of the guidelines (20/61) considered the 3-in-1 syringe as an example of AGP. Furthermore, 16 guidelines recommended around 10–30 min of fallow times, while others advised a longer time, depending on different factors.

## 4. Discussion

Generally, the primary objectives of the guidelines are to increase the standard of patient care, decrease variation, and ensure that evidence is delivered effectively [11]. Guidelines combine these features to create consensus on best practices based on the most recent evidence. This is required because patients and healthcare professionals often interpret the quality of different evidence differently [12]. Guidelines are utilized through generating recommendations to help practitioners and patients make choices regarding the most appropriate course of treatment in particular clinical circumstances [13]. This is reflected in the story of Ignaz Semmelweis, the “father of infection control,” who established handwashing regulations after learning that hand hygiene procedures could minimize the outbreak of puerperal fever [14].

Guidelines are also considered one of the cornerstones for behavioral change interventions, as they provide preparations, plans, implementation, and evaluation that aim to improve population health and wellbeing. More specifically, guidelines are intended to be the source of information for healthcare workers that focuses on addressing behavioral aspects [15]. This should be supplemented by a multifaceted approach, improving staff capabilities, opportunities, and motivations to implement the best practice stated in any guideline [16].

Looking at the results of this systematic review, variability within guidelines was detected in several domains, with risk assessment being one of them. Having an active risk assessment protocol lowers the risk of COVID-19 infection, reduces anxiety, and assures the safety and wellbeing of both patients and healthcare workers. This has been verified by the World Health Organization [17]. An example of an effective risk assessment measure that was mentioned by some guidelines is staff shielding (the permission to stay home), which was implemented for extremely vulnerable staff members who would have been put in critical status if exposed to COVID-19 [18].

One main factor showing variability in IPC practice in dentistry during COVID-19 was the emergence of different recommendations on the use of respirators. While some countries did not use respirators, others used different types with varying filtration efficacy. For example, in some countries, the FFP3 respirator was advised, which has 98% filtration rate. Others followed recommendations on using the FFP2 respirator, which has an infiltration rate of 94%. Additionally, both the N95 respirator (95% filtration rate) and the KN95 were advised in different guidelines [19]. The filtration rate is dependent on the size of aerosols because it measures mask ability to trap particles of a specific size. Because guidelines presented with divergence in aerosol, droplet, and splatter size definitions, filtration efficacy for masks varied internationally, hence the different recommendations on mask usage [20].

Another factor that led to variability in IPC practice in dentistry during the period of COVID-19 was the ventilation recommendation. The likelihood of COVID-19 dissemination increases if a patient with the infection is present in an area with inadequate ventilation. Moreover, the infection might linger in the air for a while after an infected individual has left. Having mechanical ventilation with appropriate air changes per hour is essential; however, this was not implemented worldwide [21]. It is noteworthy that adhering to the best practice of achieving 10 air changes per hour (ACH) through effective mechanical ventilation systems is crucial. Implementing 10 ACH is considered a highly effective measure in reducing air contamination and minimizing the risk of COVID-19 transmission within dental settings.

Besides ventilation, the variation in COVID-19 testing protocols for staff and patients was another factor leading to variability in IPC practice in dentistry. Multiple recommendations advised undertaking a lateral flow test for staff twice a week (every 3–4 days), and several other guidelines advised for it to be taken only once a week, while others recommended testing only when feeling symptoms [22]. It is important to note that the most infective period of COVID-19 is often the 2 days before the onset of symptoms [3]. This underscores the significance of more frequent testing, such as the recommended twice-a-week schedule, as it helps detect potential infections during the highly contagious presymptomatic phase. This approach aims to enhance the early identification of cases and contribute to the overall effectiveness of infection prevention and control measures in dental settings.

Further divergence was noted in a few areas, the definition of AGP being one of them. That is because 22 guidelines did not define AGP, and six did not elaborate on the size of aerosol particles. It is crucial to highlight that aerosol, defined as particles with a size of less than 5 *μ*m, play a significant role in the transmission of respiratory viruses, including SARS-CoV-2 [6]. Understanding the size of aerosol particles is essential, as smaller particles can remain suspended in the air for longer periods, increasing the risk of airborne transmission. Studies indicate that SARS-CoV-2 can linger in the air for extended durations, especially in enclosed spaces with poor ventilation. These airborne particles can potentially remain infectious for hours, emphasizing the importance of effective infection prevention measures in dental settings. Furthermore, recommendations on fallow time differed between some guidelines, with 16 recommending around 10–30 min, while 12 advised a longer fallow time depending on different factors such as type of procedures and number of patients in the waiting room.

Such observations underscore the diversity in the content and quality of guidance provided by various institutions in addressing IPC guidance during the spread of SARS-CoV-2 within the field of dentistry. The reasons for the variations in the guidelines arise from the decentralized nature of public health responses and the unique circumstances faced by individual nations. Different countries, grappling with distinct healthcare infrastructures, epidemiological profiles, and resource availabilities, formulated guidelines tailored to their specific contexts. Consequently, DHCWs worldwide navigated a complex landscape of recommendations. The divergence in instructions resulted in a range of health outcomes, with some nations achieving robust control measures and others facing challenges in maintaining consistent healthcare standards.

The varied impact underscores the importance of fostering international collaboration, data sharing, and the development of standardized guidelines to ensure a more equitable and effective global response to public health crises like the COVID-19 pandemic. The establishment of united guidance from a single global organization will effectively guarantee standardized and superior guidance to promote equitable recommendations and will raise dental clinical standards when future health challenges are faced.

## 5. Conclusion

This systematic review has critically evaluated and compared 61 national and international published recommendations relating to IPC in dentistry during the period of the COVID-19 outbreak. The findings of this review showed that there was divergence of recommendations and inequality across guidelines in relation to COVID-19 IPC for dentistry. This is deduced from the disparity of the AGREE II scores across the 61 guidelines and the variability in consensus scorings. This in turn emphasizes the need for an international fast-acting organization for dental guidelines, including IPC, to be responsible for developing high-quality clinical guidelines relevant to dentistry and to respond to any future emergencies in the different dental settings (dental hospitals, outpatient clinics, and community dental clinics) across the world.

### 5.1. Limitation of the Systematic Review

Various recommendations such as vaccination recommendations were officially published at a later stage, after the systematic search was conducted. Additionally, updated versions of a few of the included guidelines became available after conducting this systematic review. Therefore, they were not included in the analysis. However, this systematic review achieved its intended aim by assessing the quality of guidelines issued during the height of the COVID-19 pandemic related to IPC and the consensus rate between their recommendations.

Additionally, the evolving nature of the pandemic led to the development of guidelines by various countries at disparate intervals. Consequently, the factor of “time”emerged as a potential confounder when integrating these guidelines. This is because recommendations formulated in the initial months of the pandemic would differ from those established a year or two later, reflecting the increased understanding of the virus's transmission patterns over time.

## Figures and Tables

**Table 1:** Numerical identification of included guidelines, guideline characteristics, consensus with the gold standards, and AGREE II scores.

In line with the benchmark: 24 guidelines (representing 40% of total guidelines)						
Guideline	Date of publication	Issuing country	Issuing organization	Total consensus with the gold standard ^*∗*^	AGREE II score (%)	Reference of guideline
1	October 2020	England	National Health Service	(19/19) 100%	51	National Health Service (2020). “Standard operating procedure. Transition to recovery. (A phased transition for dental practices towards the resumption to full range of dental provision).” Version 4. https://www.nhs.uk
2	June 2020	England	Royal College of Surgeons	(3/19) 15.7%	44	Royal College of Surgeons (2020). “Recommendation for special care dentistry during the recovery phase of the COVID-19 pandemic.” https://www.rcseng.ac.uk
3	June 2020	England	Royal College of Surgeons	(3/19) 15.7%	53	Royal College of Surgeons (2020). “Recommendation for orthodontics dentistry during the recovery phase of the COVID-19 pandemic.” https://www.rcseng.ac.uk
4	July 2020	USA	Centers for Disease Control and Prevention	(3/19) 15.7%	39	Centers for Disease Control and Prevention (2020). “Interim infection prevention and control recommendations for healthcare personnel during the coronavirus disease 2019 (COVID-19) pandemic.” https://www.cdc.gov
5	June 2020	England	Royal College of Surgeons	(2/19) 10.5%	47	Royal College of Surgeons (2020). “Recommendations for Orthodontics during COVID-19 pandemic.” https://www.rcseng.ac.uk
6	June 2020	England	Royal College of Surgeons	(2/19) 10.5%	51	Royal College of Surgeons (2020). “Recommendation for Special Care Dentistry during COVID-19 pandemic.” https://www.rcseng.ac.uk
7	June 2020	Scotland	Scottish Dental Clinical Effectiveness Programme	(11/19) 57.8%	54	Scottish Dental Clinical Effectiveness Programme (2020). “Resuming general dental services following COVID-19 shutdown. A guide and implementation tools for general dental practice or phases 2 and 3 of dental services remobilisation.” Version 1.1 12. https://www.sdcep.org.uk
8	March 2020	Wales	National Health Service	(11/19) 57.8%	41	National Health Service (2020). “Red alert phase.” https://www.nhs.uk
9	January 2020	Wales	Faculty for Dental Care Professionals	(8/19) 42.1%	44	Faculty for Dental Care Professionals (2020). “Restoration of dental service post COVID-19: framework. Restoration of dental service post COVID-19: de-escalation of red alert pandemic plan.” https://www.awfdcp.ac.uk
10	May 2020	Northern Ireland	Health and Social Care Board	(19/19) 100%	44	Health and Social Care Board (2020). “Dental care in general dental practice and urgent dental care centres during the COVID-19 pandemic.” https://www.northerntrust.hscni.net
11	October 2020	UK	Faculty of General Dental Practice	(19/19) 100%	49	Faculty of General Dental Practice (2020). “Implications of COVID-19 for the safe management of general dental practice. A practical guide.” Version 2. https://www.rcseng.ac.uk
12	August 2020	England	National Health Service	(19/19) 100%	46	National Health Service (2020). “COVID-19: Guidance for maintaining services within health and care settings Infection prevention and control recommendations.” https://www.nhs.uk
13	June 2020	England	Royal College of Surgeons	(3/19) 15.7%	45	Royal College of Surgeons (2020). “Recommendation for restorative dentistry during the recovery phase of the COVID-19 pandemic.” https://www.rcseng.ac.uk
14	June 2020	England	Royal College of Surgeons	(2/19) 10.5%	45	Royal College of Surgeons (2020). “Recommendation for oral surgery dentistry the recovery phase of the COVID-19 pandemic.” https://www.rcseng.ac.uk
15	June 2020	England	Royal College of Surgeons	(6/19) 31.5%	45	Royal College of Surgeons (2020). “Recommendation for paediatric dentistry during the recovery phase of the COVID-19 pandemic.” https://www.rcseng.ac.uk
16	June 2020	England	Royal College of Surgeons	(3/19) 15.7%	39	Royal College of Surgeons (2020). “Recommendation for Oral Surgery during COVID-19 pandemic.” https://www.rcseng.ac.uk
17	June 2020	England	Royal College of Surgeons	(3/19) 15.7%	39	Royal College of Surgeons (2020). “Recommendation for Restorative Dentistry, Prosthodontics, Endodontics and Periodontics during COVID-19 pandemic.” https://www.rcseng.ac.uk
18	January 2021	Scotland	Scottish Dental Clinical Effectiveness Programme	(19/19) 100%	62	Scottish Dental Clinical Effectiveness Programme (2021). “Rapid review of aerosol generating procedures in dentistry. Mitigation of aerosol generating procedures in dentistry. A Rapid Review.” Version 1.1. https://www.sdcep.org.uk
19	April 2020	Scotland	Health Protection Scotland	(19/19) 100%	49	Health Protection Scotland (2020). “Annex 1: Infection prevention and control in urgent dental care settings during the period of COVID-19.” https://www.hps.scot.nhs.uk
20	February 2021	Northern Ireland	Health and Social Care Board	(19/19) 100%	51	Health and Social Care Board (2021). “Preparation for the re-establishment of the general dental services operational guidance.” https://www.northerntrust.hscni.net
21	October 2020	UK	Faculty of General Dental Practice	(19/19) 100%	55	Faculty of General Dental Practice (2020). “Implications of COVID-19 for the safe management of general dental practice, Synopsis.” Version 2. https://www.rcseng.ac.uk
22	December 2020	Great Britain	British Orthodontic Society	(6/19) 31.5%	46	British Orthodontic Society (2020). “BOS COVID-19 Guide to the management of Orthodontic emergencies.” https://www.bos.org.uk
23	October 2020	Europe	European Centre for Disease Prevention and Control	(19/19) 100%	48	European Centre for Disease Prevention and Control (2020). “COVID-19 infection prevention and control for primary care, including general practitioner practices, dental clinics and pharmacy setting.” https://www.ecdc.europa.eu/en
24	June 2020	UK	Association of Dental Hospitals	(19/19) 100%	49	Association of Dental Hospitals (2020). “COVID-19: Planning return to open plan clinics: guiding principles to mitigate risk.” https://www.dentalhospitals.org.uk
25	January 2021	Canada	Royal College of Dental Surgeons of Ontario	(19/19) 100%	52	Royal College of Dental Surgeons of Ontario (2021). “COVID-19: Managing Infection Risks During In-Person Dental Care.” https://www.rcdso.org
26	October 2020	Saudi Arabia	Ministry of Health	(19/19) 100%	58	Ministry of Health (2020). “Guidance for providing dental services in governmental and private sectors during COVID-19 pandemic.” https://www.moh.gov.sa
27	August 2020	Australia	Australian Dental Association	(4/19) 21.0%	40	Australian Dental Association (2020). “ADA dental service restrictions in COVID-19.” https://www.ada.org.au
28	August 2020	Australia	Australian Dental Association	(10/19) 52.6%	43	Australian Dental Association (2020). “Decision tree for level 3 patient management.” https://www.ada.org.au
29	May 2020	New Zealand	Dental Council, Ministry of Health	(19/19) 100%	50	Dental Council, Ministry of Health (2020). “Guidelines for oral health services at COVID-19 alert level 2.” https://www.dcnz.org.nz
30	March 2021	Ireland	Health Protection Surveillance Centre	(19/19) 100%	42	Health Protection Surveillance Centre (2021). “Guidance on managing infection related risks in dental services.” https://www.hpsc.ie
31	April 2020	Zimbabwe	Zimbabwe Dental Association	(5/19) 26.3%	50	Zimbabwe Dental Association (2020). “COVID-19 dental practice recommendations/guidelines.” https://www.fdiworlddental.org
32 ^*∗∗*^	October 2020	England	Public Health England four nations guidance, National Health Service	Gold standard	75	Public Health England four nations guidance (2020). “COVID-19: infection prevention and control dental appendix.” https://www.scottishdental.org
33	May 2020	England	Office of Chief, Dental Officer England	(19/19) 100%	43	Office of Chief, Dental Officer England (2020). “A Prompt to Prepare.” https://www.nhs.net
34	June 2020	England	Royal College of Surgeons	(4/19) 21.0%	54	Royal College of Surgeons (2020). “Recommendation for Dental Diagnostic Imaging during the recovery phase of COVID-19 pandemic.” https://www.rcseng.ac.uk
35	June 2020	England	Royal College of Surgeons	(6/19) 31.5%	43	Royal College of Surgeons (2020). “Recommendations for Oral Medicine during the recovery phase of the COVID-19 pandemic.” https://www.rcseng.ac.uk
36	June 2020	England	Royal College of Surgeons	(3/19) 15.7%	39	Royal College of Surgeons (2020). “Recommendations for Oral Medicine during COVID-19 pandemic.” https://www.rcseng.ac.uk
37	June 2020	England	Royal College of Surgeons	(3/19) 15.7%	41	Royal College of Surgeons (2020). “Recommendations for Paediatric Dentistry during COVID-19 pandemic.” https://www.rcseng.ac.uk
38	November 2020	Scotland	National Health Service	(19/19) 100%	55	National Health Service (2020). “Standard operating procedures for dental teams in Scotland, moving towards a return to routine dental care.” https://www.nhs.uk
39	February 2021	Scotland	Health Protection Scotland	(19/19) 100%	47	Health Protection Scotland (2020). “Novel coronavirus (COVID-19) guidance for primary care management of patients in primary care including general medical practice, general dental practice, optometry and pharmacy.” https://www.hps.scot.nhs.uk
40	April 2020	Wales	All Wales Clinical Dental Leads	(19/19) 100%	52	All Wales Clinical Dental Leads (2020). “Dental care during the COVID-19 pandemic: guidance for teams, red alert phase escalation.” https://www.dental-referrals.nhs.wales
41	July 2020	Wales	Faculty for Dental Care Professionals	(11/19) 57.8%	57	Faculty for Dental Care Professionals (2020). “Standard Operating Procedure (SOP) to Inform Orthodontic Treatment in Wales During COVID-19 pandemic.” https://www.awfdcp.ac.uk
42	June 2020	Wales	Faculty for Dental Care Professionals	(19/19) 100%	44	Faculty for Dental Care Professionals. “Standard Operating Process for Non-COVID-19 Dental Centres Providing Aerosol Generating Procedures in Wales.” https://www.awfdcp.ac.uk
43	July 2020	Northern Ireland	Health and Social Care Board	(4/19) 21.0%	45	Health and Social Care Board (2020). “Urgent dental care flow diagram.” https://www.hscboard.hscni.net
44	May 2020	Canada	Canadian Dental Association	(19/19) 100%	35	Canadian Dental Association (2020). “Adapting the dental office to the COVID-19 pandemic, return-to-practice office manual.” https://www.cda-adc.ca
45	April 2020	Saudi Arabia	Ministry of Health	(11/19) 57.8%	44	Ministry of Health (2020). “Dental emergency protocol during COVID-19 pandemic.” https://www.moh.gov.sa
46	August 2020	Australia	Australian Dental Association	(19/19) 100%	58	Australian Dental Association (2020). “ADA COVID-19 Risk Management Guidance.” https://www.ada.org
47	August 2020	Australia	Australian Dental Association	(7/19) 36.8%	45	Australian Dental Association (2020). “Decision tree for level 2 patient management.” https://www.ada.org
48	March 2021	New Zealand	Dental Council, Ministry of Health	(11/19) 57.8%	38	Dental Council, Ministry of Health (2021). “Guidelines for oral health services at COVID-19 Alert Level 3.” https://www.england.nhs.uk/coronavirus/wp-content/uploads/sites/52/2020/06/C0581-covid-19-urgent-dental-care-sop-update-16-june-20-.pdf
49	April 2020	Poland	Polish Dental Association	(11/19) 57.8%	52	Polish Dental Association (2020). “The Polish dental association working group recommendations for procedures in dental office during an increased epidemiological risk.” https://www.fdiworlddental.org/polish-dental-society
50	August 2021	United Arab Emirates	Dubai Health Authority	(19/19) 100%	52	Dubai Health Authority (2020). “Guidelines for the provision of dental services during COVID-19.” https://www.dha.gov.ae
51	June 2020	Australia	Australian Dental Association	(11/19) 57.8%	44	Australian Dental Association (2020). “ADA environmental cleaning and disinfection guidance for dental practitioners in the context of COVID-19.” https://www.ada.org
52	July 2020	USA	American Dental Association	(8/19) 42.1%	53	American Dental Association (2020). “Return to work interim guidance toolkit.” https://www.ada.org
53	May 2020	China	Chinese Stomatological Association	(9/19) 47.3%	45	Chinese Stomatological Association (2020). “Chinese response to COVID-19 in dental field.” https://www.fdiworlddental.org
54	June 2020	England	Royal College of Surgeons	(4/19) 21.0%	41	Royal College of Surgeons (2020). “Recommendations for diagnostic imaging during COVID-19 pandemic.” https://www.rcseng.ac.uk
55	October 2020	Australia	Australian Dental Association	(7/19) 36.8%	23	Australian Dental Association (2020). “Decision tree for level 1 patient management.” https://www.ada.org
56	January 2020	Australia	Government of New South Wales	(9/19) 47.3%	53	Government of New South Wales (2020). “COVID-19 guidelines for public dental services.” https://www.health.nsw.gov.au/oralhealth/Factsheets/covid19-guide-public-dental-services.pdf
57	August 2020	New Zealand	Dental Council, Ministry of Health	(11/19) 57.8%	50	Dental Council, Ministry of Health (2020). “Guidelines for oral health services at COVID-19 Alert Level 1.” https://www.england.nhs.uk/coronavirus/wp-content/uploads/sites/52/2020/06/C0581-covid-19-urgent-dental-care-sop-update-16-june-20-.pdf
58	May 2020	Philippines	Philippine Dental Association	(19/19) 100%	53	Philippine Dental Association (2020). “Updates on the PDA interim guidelines on infection prevention for COVID-19 Pandemic.” https://www.fdiworlddental.org
59	October 2020	England	National Health Service	(5/19) 26.3%	48	National Health Service (2020). “COVID-19 guidance and standard operating procedure for the provision of urgent dental care in primary care dental settings and designated urgent dental care provider sites.” https://www.nhs.uk
60	April 2020	Great Britain	Public Health England, Academy of Medical Royal Colleges, Public Health Wales, Health protection Scotland, Public Health Agency, National Health Service	(5/19) 26.3%	38	Public Health England, Academy of Medical Royal Colleges, Public Health Wales, Health protection Scotland, Public Health Agency (2020). “Recommended PPE for primary, outpatient, community and social care by setting, NHS and independent sector.” https://assets.publishing.service.gov.uk/media/5ea6b31686650c031b442238/Table_2._Recommended_PPE_for_primary__outpatient__community_and_social_care.pdf
61	September 2021	India	Ministry of Health and Family Welfare	(19/19) 100%	49	Ministry of Health and Family Welfare (2021). “National guidelines for safe dental practice during COVID-19 pandemic.” https://www.mohfw.gov.in/pdf/NationalGuidelinesforSafeDentalPracticeDuringCovid19pandemic.pdf

COVID-19, coronavirus disease 2019; BOS, British Orthodontic Society; ADA, Australian Dental Association; SOP, Standard Operating Procedure; PDA, Philippine Dental Association; PPE, personal protective equipment; NHS, National Health Service.  ^*∗*^Total consensus score: The total score and percentage of guidelines being in line with the benchmarking recommendations.  ^*∗∗*^Gold standard guidelines according to AGREE II tool.

**Table 2:** Summary of benchmarking recommendations, consensus scores of the guidelines, and the main reasons for variability.

Domain	Benchmark recommendations ^*∗*^ (version: October 2020)	Domain consensus ^*∗∗*^	Reason for variability
Definition			
AGP	Any medical, dental, or patient care procedure that can result in the release of airborne particles <5 *μ*m in size from the respiratory tract of an individual	45.9% (28/61)	Not mentioned; examples were given without a definition; no reference to airborne particles nor their size
Non-AGP	Not mentioned in the version included in this systematic review	16.3% (10/61)	Not mentioned; examples were given without a definition

Protocol for PPE			
For AGP	Disposable gloves (not vinyl), disposable gown, FFP3, eye/face protection	78.6% (48/61)	Not mentioned; different respirators were recommended (other than FFP3 or FRSM)
For non-AGP	Disposable gloves (not vinyl), disposable plastic apron, FRSM, and eye/face protection	57.3% (35/61)	Not mentioned.

Fallow time after AGP	Dependent on-air changes per hour and procedural mitigating factors but does not fall below 10 min for patients in medium- or high-risk pathways	45.9% (28/61)	Not mentioned; different time given (e.g., 1 hr).

Ventilation requirement	Mandatory for all AGPs. Whole building ventilation should be 10 L/s/person and treatment room should have at least 10 air changes per hour (ACH)	52.4% (32/61)	Not mentioned; different ACH; or mentioned but with no specific period recommended

Triaging recommendation	Screening and triaging must be undertaken prior to the patient attending the dental setting/hospital or immediately upon arrival	88.5% (45/61)	Not mentioned; one pathway for all patients regardless of their risk

Telephone clinic	Not mentioned in the version included in this systematic review	55.7% (43/61)	Not mentioned

Protocol for risk assessment			
For staff	Should be assessed by employers and manage their work commitments accordingly	44.2% (27/61)	Not mentioned
For patients	Divided into high, medium, and low risk with details on PPE and RPE for each group	57.3% (35/61)	Not mentioned; one pathway for all patients regardless of their risk

Measures for vulnerable patients	Not mentioned in the version included in this systematic review	42.6% (26/61)	Not mentioned; diseases not listed

Hard-surface disinfection regimen	Disinfectant agent such as chlorine at 0.1% or 1,000 ppm or an equivalent against viruses, bacteria, and fungi to EN standard 14,476 for viricidal activity should be used	59.0% (36/61)	Not mentioned; not detailed (only mentioning the use of disinfectant wipes without percentage of ethanol or hydrogen peroxide)

Hand hygiene			
Mentioned or not	Yes	60.6% (37/61)	Not mentioned
Level	Hands should be washed with soap and water: use of at least 60%–80% alcohol-based hand rub before and after caring for/treating a patient, entering and leaving the surgery, and after removal of PPE	22.9% (20/61)	Not mentioned; description not given

Type of respirator recommended	FFP3 for AGPs	72.1% (44/61)	Not mentioned; listed different types than FFP3

Protocol for COVID-19 positive patients	TBPs are additional measures to SICPs required when caring for patients/individuals with a known or suspected infection such as COVID-19	72.1% (44/61)	Not mentioned; listed under high-risk, but no protocol described for such patients

Test requirement			
For staff	Staff should not come to work if symptomatic until negative test results are available	19.6% (12/61)	Not mentioned; not compulsory
For patients	There must be screening/triaging/testing of patients for infection risk	32.7% (20/61)	Not mentioned

Vaccination recommendation for staff	No recommendations as vaccines were under development at that stage	11.4% (7/61)	Not mentioned; prior to vaccination development

AGP, aerosol-generating procedures; PPE, personal protective equipment; COVID-19, coronavirus disease 2019; ACH, air changes per hour; RPE, respiratory protective equipment; TBPs, transmission-based precautions. ^*∗*^Benchmark recommendations: The recommendations published by the gold standard on each domain. ^*∗∗*^Consensus score: The number and percentage of different recommendations agreeing with the gold standard in the specified.
